# Densely Connected Networks with Multiple Features for Classifying Sound Signals with Reverberation

**DOI:** 10.3390/s23167225

**Published:** 2023-08-17

**Authors:** Zhuo Chen, Dazhi Gao, Kai Sun, Xiaojing Zhao, Yueqi Yu, Zhennan Wang

**Affiliations:** Department of Marine Technology, Ocean University of China, Qingdao 266100, China; chenzhuo3286@stu.ouc.edu.cn (Z.C.); sunkai3862@stu.ouc.edu.cn (K.S.); zhaoxiaojing@stu.ouc.edu.cn (X.Z.); yyq9011@stu.ouc.edu.cn (Y.Y.); wangzhennan@stu.ouc.edu.cn (Z.W.)

**Keywords:** multilevel feature, reverberation, densely connected network, convolutional neural network

## Abstract

In indoor environments, reverberation can distort the signalseceived by active noise cancelation devices, posing a challenge to sound classification. Therefore, we combined three speech spectral features based on different frequency scales into a densely connected network (DenseNet) to accomplish sound classification with reverberation effects. We adopted the DenseNet structure to make the model lightweight A dataset was created based on experimental and simulation methods, andhe classification goal was to distinguish between music signals, song signals, and speech signals. Using this framework, effectivexperiments were conducted. It was shown that the classification accuracy of the approach based on DenseNet and fused features reached 95.90%, betterhan the results based on other convolutional neural networks (CNNs). The size of the optimized DenseNet model is only 3.09 MB, which is only 7.76% of the size before optimization. We migrated the model to the Android platform. The modified model can discriminate sound clips faster on Android thanhe network before the modification. This shows that the approach based on DenseNet and fused features can dealith sound classification tasks in different indoor scenes, and the lightweight model can be deployed on embedded devices.

## 1. Introduction

The development of active noise-canceling headphones and hearing aids has resulted in users demanding increasingly high levels of performance from these devices. One of the development directions of active noise cancelation technology is to achieve noise reduction or speech enhancement through sound classification techniques to distinguish the sound environment the user is in and improve the user’s listening experience. Sound classification techniques are widely used in spoken language recognition [1], environmental sound classification [2], music genre classification [3], heart sound classification [4], and breathing sound classification [5].

When indoors, reverberation from the internal structure of a house can alter the original sound signal, creating delays and causing the distortion of the corresponding features. Most of the datasets used to train sound classification models do not have reverberation effects. Therefore, the models trained from these datasets will be less accurate when applied to indoor environments. We therefore considered the indoor application of hearing aids and active noise-canceling headphones and set our classification targets as music signals, specifically instrumental music, song signals that contain both instrumental music and human voices, and speech signals that contain only human voices.

When training sound classification models, the time-domain signal of the sound is usually made into artificial features, such as the mel frequency cepstral coefficient (MFCC) [6], perceptual linear predictive (PLP) coefficient [7], the linear prediction coefficients (LPC) [8], and the linear prediction cepstral coefficient (LPCC) [9], which are used as inputs to the classification models.

A single artificial feature implies incomplete information. Therefore, combining these features to improve the classification model’s accuracy is a hot topic in the research on sound classification problems. In [10], Li combined features such as the MFCC, fundamental frequency, spectral center of mass, and sub-band energy as input feature vectors. In [11], the discrete wavelet transform (DWT) and MFCC features were combined to improve the accuracy of the classification of heart sounds.

Due to the powerful performance of CNNs in image processing, spectrograms can also be used for sound classification [12]. In [13], spectrograms were fed into a CNN-LSTM to distinguish between speakers with face masks. In [14], optimized S-transform OST features based on different scales were combined and fed into a CNN model to perform the triple classification of breath sounds. In [15], researchers used mel spectrograms as data and combined self-focus and multilayer feature extraction methods to classify normal breath sounds. Similar to residual networks (ResNet) [16], DenseNet establishes close connections between layers, allowing the training of deeper models [17]. The better performance of DenseNet makes it widely used for sound classification. In [18], DenseNet showed good performance in lung sound classification. In [19], the dense module in DenseNet was used to perform feature extraction on sound data. In [20], the mel spectrogram and log-mel spectrogram were fed to a CNN to automatically diagnose cardiovascular diseases (CVDs). In [21], continuous wavelet transform (CWT), mel spectrogram, and Gammatone spectrogram data were combined into a 3D channel spectrogram for the automatic detection of speech and phoneme class recognition to extract telephone attribute features.

**Figure 1:**
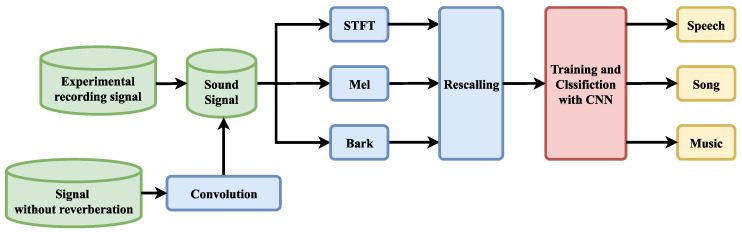
Flowchart of sound classification based on fused features and deep residual network.

In this paper, we propose a classification method based on fusion features and DenseNet to distinguish music signals, song signals, and speech signals under the reverberation effect and make a reasonable reverberation dataset to test the method. Reverberation introduces distortion into the signal. This distortion can be reflected in the spectrogram. As in the case of speech signals and song signals, the audio features are lengthened and intuitively look more similar to the spectrogram of a musical signal. In addition, different rooms correspond to different impulse responses, causing the distortion from reverberation to be different. On this basis, the dataset in this paper was produced with sound signals with different reverberation effects using the impulse responses of several rooms. As shown in Figure 1, these signals are combined with the recorded sound signals to form the dataset. Therefore, this is a challenging dataset. We propose combining the short-time Fourier transform (STFT), mel, and bark data to form a multichannel spectrogram. These three spectrograms are from different frequency scales and have more features than single-scale spectrograms. We modified DenseNet to make it more lightweight. In the proposed method, the fused features are used as the input to DenseNet for classification. The challenging dataset used in this study includes sound signals with multiple reverberation effects, and DenseNet was trained and tested on this dataset.

## 2. Proposed Method

In this study, we trained the model on DenseNet by fusing STFT, bark, and mel features to distinguish three sound signals: music, songs, and speech. A classification flowchart based on the improved DenseNet and fused features used in this paper is shown in Figure 1. First, feature extraction was performed on the time-domain signal to obtain three speech spectrogram features based on different frequency scales to compose fusion features. After the training and classification processes of DenseNet were completed, the three signal sets were recognized. The extraction method and combination method of the three features are described in Section 2.1. The training and classification processes using DenseNet is described in Section 2.2. Section 2.3 briefly introduces concepts related to reverberation and shows the effect of reverberation on the features.

### 2.1. Feature Extraction and Fusion

In convolutional neural network-based sound classification, spectrogram features are often used as input to the training model. Since the STFT, bark, and mel features are extracted based on different frequency-domain scales, each feature contains unique information. To make the information extracted from the time-domain signal more comparable, we used the same frame length and step size to ensure that the features were aligned on the time axis and to facilitate the subsequent training.

#### 2.1.1. STFT

STFT reflects the frequency change in the signal over time at standard frequencies and is one of the most commonly used features for sound classification. The basic idea of STFT is to truncate the signal in the time domain by a window function and treat the signal in the window as a smooth signal, then perform a Fourier transform on the intercepted signal in the window to finally obtain a two-dimensional time–frequency matrix.

In this paper, the frequency dimension of the STFT feature was set to 256, and the sampling frequency of the sound signal used in this paper was 8192 Hz, resulting in a frequency resolution of 16 Hz.

#### 2.1.2. Mel Scale

Considering that the response of the human ear to frequency is nonlinear, we converted the frequency domain of the sound signal from the standard scale to the mel scale. Mel filter banks were used to delineate the critical bands and extract the feature coefficients to represent the human ear’s perception of isometric pitch changes [22]. In the past, due to the limitations of machine learning algorithms, highly correlated features such as mel features needed to undergo a discrete cosine transform to extract MFCC features from mel features, and then the MFCC was input into a model such as the Gaussian mixture model–hidden Markov model (GMM-HMM) [23]. Since the discrete cosine transform is linear, a considerable portion of nonlinear information was lost in this process. In recent years, with the development of deep learning in speech and the fact that deep neural networks are not easily affected by highly correlated inputs, people have started to use mel features as input data for models [24]. In this paper, the range of the mel scale was set to 128.

**Figure 2:**
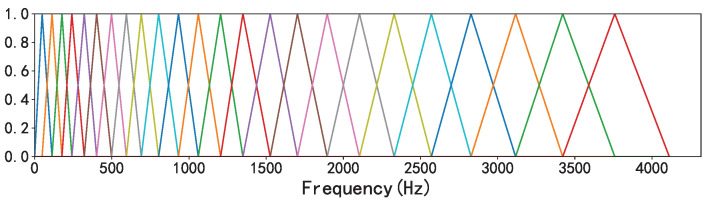
Mel filter banks.

Figure 2 shows the mel filter bank when the sample rate was set to 8192 Hz and the number of filters was 32.

#### 2.1.3. Bark Scale

The bark scale is a psychoacoustic scale that uses a trapezoidal filter bank to map standard frequencies to the 24 critical frequency bands of psychoacoustics [25,26]. Similar to the relationship between mel and the MFCC, the bark filter bank is the preprocessing step to extract PLP features.

**Figure 3:**
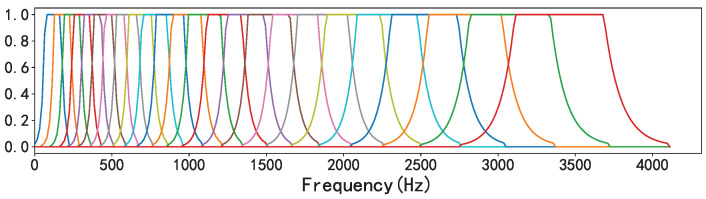
Bark filter banks.

Figure 3 shows the bark filter bank when the sample rate was set to 8192 Hz and the number of filters was 32. In this paper, the range of the bark scale was set to 128.

#### 2.1.4. Fusion

**Figure 4:**
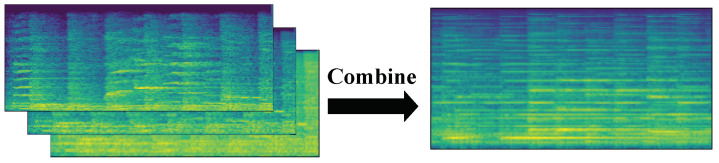
Feature fusion methods.

In this paper, a fusion method based on feature extraction was used to integrate multiple features into a new feature, and then the integrated feature set was fed into a neural network or classifier. Figure 4 illustrates the process of combining the three spectrogram features into three data channels.

The time-domain signal was first divided into equal-sized lengths, and equal windows and steps were used for each segment of the time-domain signal in feature extraction to ensure the consistency of the time scale. Here, the duration of each signal was 4 s, the sampling rate was 8192 Hz, the selected window length was 512, and the step size was 128. The final extracted STFT image size was 257 × 257, the mel feature size was 128 × 257, and the bark feature size was 128 × 257. the size of these three features was uniformly resized to 128 × 256 using the OpenCV image processing library. To reduce the quantity of data and to facilitate computations, each 128 × 256 matrix was deflated into the interval 0 to 255. Each matrix was then rounded to the nearest whole number, and the data type of such a sample was converted from float32 to uint8. These fused features were fed into the convolutional neural network for training.

Spectrogram fusion was performed by combining three spectrograms, i.e., three two-dimensional arrays, in a third dimension. Similar to the RGB channels of a color picture, here, we enter one spectrogram per channel.

### 2.2. DenseNet

Many efforts were made to overcome overfitting. The emergence of ResNet makes it possible to increase the number of layers to make the network more capable. The main advantages of DenseNet include that it requires fewer parameters and demonstrates good regularity [17]. Considering these advantages, we input the fused features into DenseNet for training.

Considering the application scenario of active noise reduction and the characteristics of fused features, some adjustments were made to the structure of the DenseNet model, and the modified model was named DenseNet-v1. As shown in Table 1, only four convolutional layers were set in each dense block to make the model lightweight. In addition, the preprocessing layer was modified from a 7 × 7 convolutional layer with a step size of 2 to a 3 × 3 convolutional layer with a step size of 1, and the fill amount was modified from 3 to 1.

### 2.3. Reverberation

When sound waves travel indoors, they are reflected many times by different materials and housing structures, and the sound that reaches the human ear can be divided into direct sound and reflected sound. As a result, the reverberated sound sounds longer. The reverberation time is the time it takes for the sound reflected in the space to decay by 60 dB after the source sound stops. Because of the material absorption properties of different frequency bands, the reverberation time in each band is also different, and these reverberation times together are called the reverberation time curve.

The multipath effect occurs when one attempts to transmit and receive an audio signal in a relatively narrow area. That is, there is more than one path from the sound source to the reception point. Depending on the path of sound propagation, these paths can be categorized into paths made up of direct and nondirect sound waves. The number of reflections and propagation paths of these nondirect waves each occurring in the room are different, and each reflection will lead to a certain degree of amplitude attenuation and phase delay.

**Figure 5:**
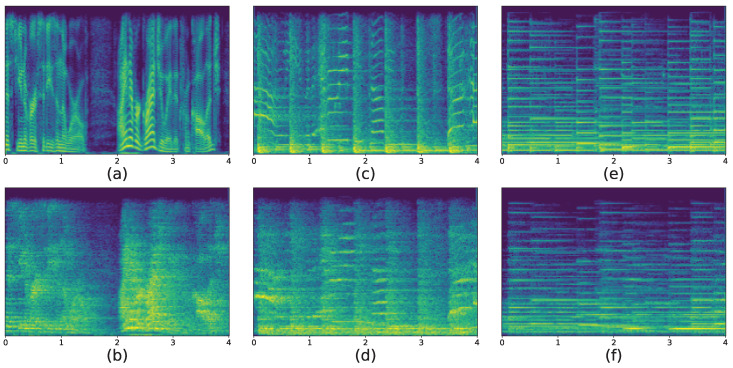
The effect of reverberation on the STFT spectrogram. (**a**,**b**) are the spectra of the speech signal without and with the reverberation effect, respectively. (**c**,**d**) are the spectra of the song signal without and with the reverberation effect, respectively. (**e**,**f**) are the spectra of the music signals without and with the reverberation effect, respectively.

The reverberation effect in the time-domain signal is demonstrated in the spectrogram, making the continuation of the band energy longer so that the syllable becomes blurred. As an example, the STFT characteristics of three sound signals are shown in Figure 5, where the STFT of a sound signal recorded in the studio is compared with the STFT of this sound after playback and recording in the room.

As seen in Figure 5, the special structures of song and speech signals are blurred due to the effect of reverberation, and the energy of each frequency band becomes longer in the time domain and appears to be shaped similarly to music signals from an intuitive visual point of view.

Data can also be obtained by means of simulation in addition to recording sound directly in the room to obtain a signal with the reverberation effect. There are three types of digital artificial reverberation methods that are commonly used: impact response convolution methods, computational acoustic methods, and delay network methods. Convolution methods convolve a clean and reverberation-free signal with the room’s impact response. Reilly proposed this method in 1995 [27]. Lehman [28] et al. proposed a mirror source model spectrum based on the impulse response of the room.

In the room, the source signal $s_{0}\left(t\right)$ is set to arrive at the receiving microphone after multipath propagation, and the received signal $s\left(t\right)$ can be expressed as

(1) $$s\left(t\right)=s_{0}\left(t\right)*h\left(t\right)+n\left(t\right)$$

where $h\left(t\right)$ denotes the room impulse response between the sound source and the microphone, ∗ is the sign of the convolution operation, and $n\left(t\right)$ is the noise signal.

The reverberation time [29] is the most important reverberation parameter; it can be understood as the duration of reverberation and reflects the strength of the reverberation effect. The reverberation time is defined as the time elapsed from the emission of the sound from the source to the stabilization of the reverberant environment in the room. It is often expressed by $RT_{60}$. In different acoustic scenarios, there are different requirements for reverberation. For example, $RT_{60}$ in classrooms is adjusted to 0.5~0.9 s, which is higher than the value of 1.8 s considered for concert halls. In contrast, for regular indoor call scenes, $RT_{60}$ is usually required to be lower than 0.6 s. For speech recognition devices trained with conventional speech, the reverberation time should preferably be controlled within 0.2 s.

## 3. Dataset Production

**Figure 6:**
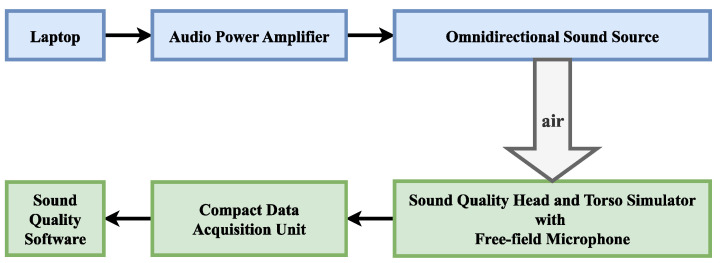
Experimental apparatus connection method.

In this section, the process of making a dataset is described. Datasets with reverberation effects are prepared by live recordings and simulations. We conducted the experiments in December 2022 in the acoustic hall of the Laoshan campus of the Ocean University of China. To ensure experimental accuracy, the key instrument used in the experiment was the B&K instrument. The specific experimental instrument models are shown in Table 2 below. The connections of these devices are shown in Figure 6.

Before starting the experiment, the free-field microphone was calibrated using a sound calibrator. The calibrator produces 94 dBSPL at 1 kHz, allowing the acoustic transducer to be calibrated at this standard. The sample rate was set to 8192 Hz. The receiver was placed in the center of the acoustic hall, and the ambient noise at the receiver was measured by a sound level meter when audio was not being played. The sound pressure level of the ambient noise was approximately 30 db SPL. The speakers were placed 10 m away from the receiver. The audio signals were recorded for 200 s for each type of audio signal by playing and recording three types of audio data in the room: music, songs, and speech.

To expand the dataset and to improve the robustness of the model, a dataset with reverberant features was produced using a simulation method. The clean audio data were convolved with the impulse response of the room using the time-domain signal convolution method to simulate the effect of the sound signal playing in the room.

**Figure 7:**
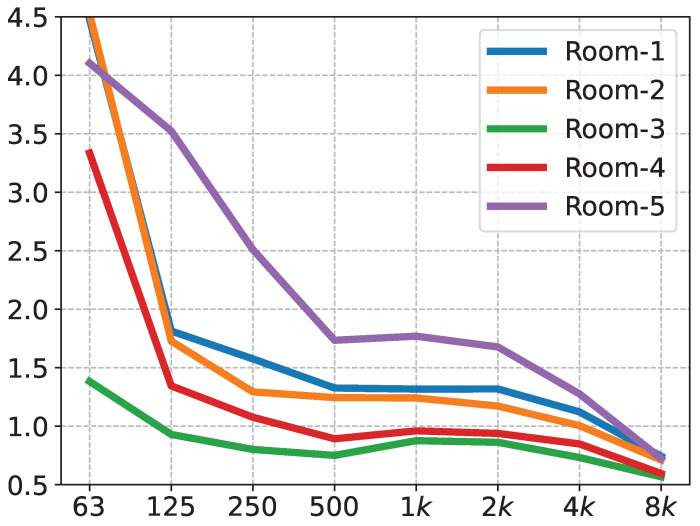
Reverberation time curve.

The DIRAC room acoustics software and its accompanying equipment were used to measure the reverberation time; the sound source was a firing gun. Table 2 shows the main instruments used in the experiments, as well as the software. The experimental procedure was as follows: the measurement personnel holding the firing gun held the gun above their heads at a distance of approximately 2 m from the horizontal plane. The measurement points were greater than 1 m from each reflecting surface. In total, the impulse responses were recorded in five rooms, and their corresponding reverberation time curves are shown in Figure 7.

Rooms 1 and 4 are a stepped classroom and a regular classroom, respectively. Rooms 2 and 3 are acoustically designed stepped classrooms and general classrooms, respectively, which were retrofitted with sound-absorbing structures, such as perforated panels. Room 5 is a music classroom. Typically, the reverberation time requirements for different room functions are reflected in the midfrequency (average between 500, 1000, and 2000 Hz) reverberation times [30]. The midfrequency reverberation times of Room 3 and Room 4 fulfill the requirements to hold a general verbal conversation. Room 1 and Room 2 have midfrequency reverberation times adequate for general singing scenarios. Room 5 has a midfrequency reverberation time that meets the general concert hall reverberation time requirements. These five rooms represent, as much as possible, the scenarios that will be encountered in life.

**Figure 8:**
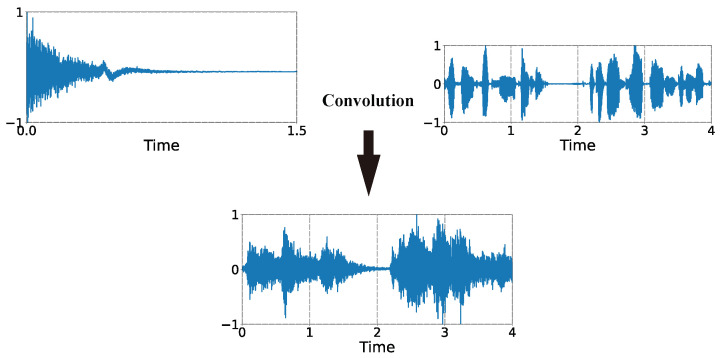
Convolution of signal and impulse responses to obtain the reverberation effect.

Acoustic measurements were made in these rooms, and the corresponding impulse responses were obtained for each room. As shown in Figure 8, these impulse responses were convolved with the acoustic signal to obtain the acoustic signal with the reverberation effect. Since the reverberation time curves of these five rooms have their own characteristics, they can represent the different rooms in which we performed sound playback and recording. Therefore, the reverberation dataset we simulated based on the impulse response of these rooms is robust.

**Figure 9:**
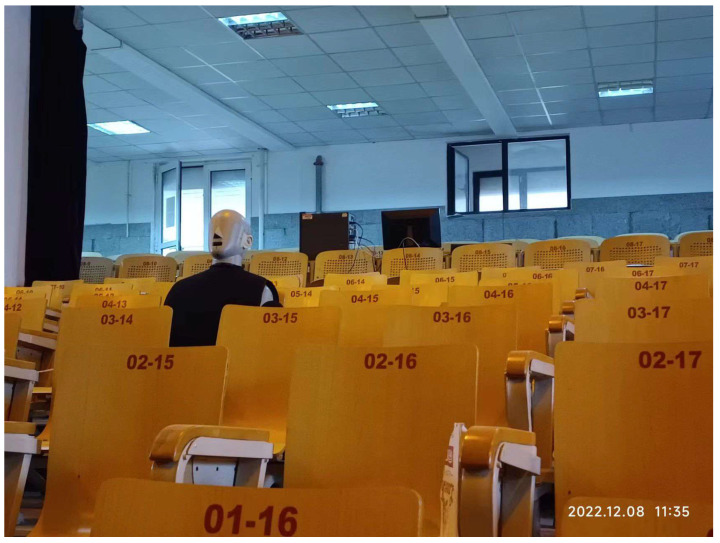
On-site experiments.

As shown in Figure 8, the measured impulse responses were convolved with the clean signals in the time domain to obtain signals with reverberant characteristics. Figure 9 was taken in the field experiment, and the instruments shown are the sound quality head and torso simulator. The dataset consists of the signals recorded in the hall and the signals obtained from the simulation; both types of signals were included to make our dataset more diverse.

We chose a time window of 4 s and a step size of 2 s. A total of 294 samples were extracted from the recorded audio. For each sound signal, 200 samples were selected to prepare the dataset. By processing each of the five impulse responses with these time-domain signals, 3000 samples were obtained. Each of these samples was 4 s long. They were combined into a fused feature dataset according to the method described in Section 2.1.4.

In this paper, the training, validation and test data were divided according to the ratio of 6:2:2. In summary, we trained the model with approximately 66 min of audio, validated the model with 22 min of audio, and tested the model with 22 min of audio.

## 4. Results

To evaluate the effectiveness of the proposed fusion feature-based network model and the DenseNet-v1 network model, the Visual Geometry Group (VGG) [31], DenseNet-121, Google Inception Net (GoogLeNet) [32], and ResNet-18 models were trained on the same dataset for comparison. In this study, the models were trained using a stochastic gradient descent (SGD) optimizer with a batch size set to 64 and an initial learning rate of 0.005. The best models within 400 epochs were retained on the validation set, and the accuracy of these models was tested on the test set. All networks were implemented in PyTorch. We saved the model network parameters locally after we finished training the model. We compared the models’ sizes to the network parameters. Additionally, we deployed the models on embedded devices based on each network’s parameters. The training platform was an NVIDIA TESLA T4 GPU. The test platform was a Qualcomm Snapdragon 662.

We can obtain the following from Table 3. GoogLeNet had the worst accuracy. The accuracy of VGG-11 was better, but its model size of 373 MB made it difficult to migrate to embedded platforms. Similar to ResNet-18, which uses 4 convolutional layers per residual block, DenseNet-v1 also uses 4 convolutional layers per dense block, and the accuracy of DenseNet-v1 was 2.43% higher than that of ResNet-18. The accuracy of DenseNet-v1 was higher than that of DenseNet-121, and the size of the DenseNet-v1 model was only 7.76% of the size of the DenseNet-121 model, which was only 3.09 MB. This was because we reduced the dense Block structure in the model. Smaller network models were easier to migrate to embedded platforms.

**Figure 10:**
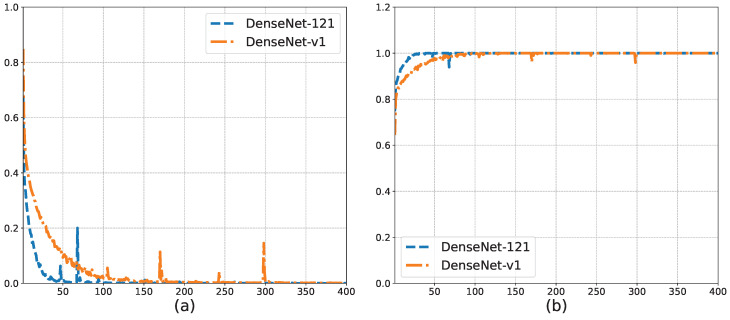
Comparison of the training process before and after DenseNet modification. (**a**) Training loss. (**b**) Training accuracy.

Figure 10a,b shows the training loss and training set accuracy of the DenseNet-v1 and DenseNet-121 model training processes based on the training set and validation set, respectively. The trend of the curves in Figure 10 shows that both models reached global convergence. In addition, compared with DenseNet-121, the training loss of DenseNet-v1 decreased more slowly, and the rate of the increase in the training set accuracy was slower, although both values were almost the same in the end. This shows that after we adjusted the network structure, the initial learning ability of the model was reduced, which is consistent with the simplified nature of the network structure. We simplified part of the network structure but weakened protections against possible overfitting simultaneously. As a result, DenseNet-v1 outperformed DenseNet-121 on the test set. We found that the peaks on the training loss curve remained after adjusting the batch size and learning rate. Therefore, we believe that this peak was caused by erroneous samples. For the song signal, when the window slid to the gap between two lyrics, the intercepted signal contained less vocal signal. Therefore, the features of this part of the signal were closer to the music signal. During the training process, when a certain batch size contained more such erroneous samples, the training loss fluctuated more widely.

**Figure 11:**
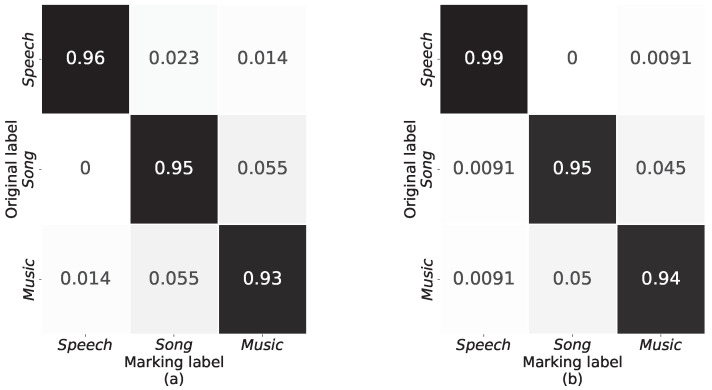
Confusion matrix. (**a**) DenseNet-121. (**b**) DenseNet-v1.

Figure 11 and Table 4 show that DenseNet-v1 outperforms DenseNet-121 on the confusion matrix, precision, recall, and F1 scores. It can be seen that DenseNet-v1 is significantly better than DenseNet-121 in recognizing sound signals with reverberation when fusion features are used as input. In addition, we reduced the dense block structure of the architecture.

We migrated the model to the Andriod platform to complete our tests and better illustrate our model’s validity. First, we converted the saved network parameters from PyTorch to an MNN. The size of the network model remained largely unchanged during this process. The platform chosen for testing was Snapdragon 662, which is an entry-level processor. As shown in Table 5, on the same test platform, the time used by DenseNet-v1 to predict a sample was only 64.56% of that of DenseNet-12.

Thus, compared with other CNNs, DenseNet-v1 can recognize speech signals, song signals, and music signals very well. In addition, due to the reduction in the model’s dense block structure, DenseNet-v1 is a small model that is still capable of making fast predictions. These characteristics make our proposed approach ideal for migrating to embedded devices.

## 5. Conclusions

Pursuing active noise reduction techniques requires a more accurate determination of the acoustic environment in which the device is located. In other words, distortion in the spectrogram is caused by scattering between the sound and obstacles during propagation. Reverberation brings difficulties and challenges to sound classification. In this paper, we propose the fusion feature and DenseNet-based differentiation of sounds with reverberation. In this paper, three spectrogram features of the STFT, mel, and bark data based on different frequency scales are extracted from the time-domain signal, and these features are scaled according to the same range and combined into three-channel data. These features are then input into the optimized DenseNet network model for training and classification. The experimental results show that the accuracy of our method is 95.90% on the dataset with reverberation features, and the size of the model is only 3.09 MB, which is much smaller than that of other CNNs. In summary, the method based on fusion features and DenseNet can reliably identify signals with reverberation features and is both lightweight and capable of fast recognition.

In the future, our research related to the method presented in this paper will be improved in the following aspects. We will try other deep learning based methods to create lighter classification models with fewer model parameters. Additionally, we will create datasets with more sound types and reverberation effects to improve the usefulness and robustness of the model.

## Figures and Tables

**Table 1:** DenseNet-121 and DenseNet-v1 architectures.

Layers	Output Size (DenseNet-121)	DenseNet-121 (k = 32)	DenseNet-v1 (k = 32)	Output Size (DenseNet-v1)
Convolution	64 × 128	7 × 7 conv, stride 2	3 × 3 conv, stride 1	128 × 256
Pooling	32 × 64	3 × 3 conv, stride 2		64 × 128
Dense Block(1)	32 × 64	$\left[\begin{array}{c} 1\times 1\;\;\mathrm{conv}\\ 3\times 3\;\;\mathrm{conv} \end{array}\right]\times 6$	$\left[\begin{array}{c} 1\times 1\;\;\mathrm{conv}\\ 3\times 3\;\;\mathrm{conv} \end{array}\right]\times 4$	64 × 128
Transition Layer(1)	32 × 64	1 × 1 conv		64 × 128
	16 × 32	2 × 2 average pool, stride 2		32 × 64
Dense Block(2)	16 × 32	$\left[\begin{array}{c} 1\times 1\;\;\mathrm{conv}\\ 3\times 3\;\;\mathrm{conv} \end{array}\right]\times 12$	$\left[\begin{array}{c} 1\times 1\;\;\mathrm{conv}\\ 3\times 3\;\;\mathrm{conv} \end{array}\right]\times 4$	32 × 64
Transition Layer(2)	16 × 32	1 × 1 conv		32 × 64
	8 × 16	2 × 2 average pool, stride 2		16 × 32
Dense Block(3)	8 × 16	$\left[\begin{array}{c} 1\times 1\;\;\mathrm{conv}\\ 3\times 3\;\;\mathrm{conv} \end{array}\right]\times 24$	$\left[\begin{array}{c} 1\times 1\;\;\mathrm{conv}\\ 3\times 3\;\;\mathrm{conv} \end{array}\right]\times 4$	16 × 32
Transition Layer(3)	8 × 16	1 × 1 conv		16 × 32
	4 × 8	2 × 2 average pool, stride 2		8 × 16
Dense Block(4)	4 × 8	$\left[\begin{array}{c} 1\times 1\;\;\mathrm{conv}\\ 3\times 3\;\;\mathrm{conv} \end{array}\right]\times 16$	$\left[\begin{array}{c} 1\times 1\;\;\mathrm{conv}\\ 3\times 3\;\;\mathrm{conv} \end{array}\right]\times 4$	8 × 16
Classification Layer	1 × 1	Global average pool		1 × 1
		3-d fc, softmax		

**Table 2:** Experimental apparatus list.

Instrument	Type
Audio power amplifier	Type 2716-C
Omnidirectional sound source	Type 4295
Sound quality head and torso simulator	Type 4100
Free-field microphone	Type 4190
Compact data acquisition unit	Type 3560-B
Sound calibrator	Type 4231
Sound quality software	Pulse (version 11.1)
Room acoustics software	DIRAC (version 6.0)

**Table 3:** Comparison of the classification accuracy and model size of different network architectures.

Model	Accuracy (%)	Model Size (MB)
GoogLeNet	83.00	23.9
VGG-11	90.74	373
ResNet-18	93.47	44.8
DenseNet-121	94.69	39.8
DenseNet-v1	95.90	3.09

**Table 4:** Comparison of the performance of DenseNet-121 and DenseNet-v1.

Model	Sound Class	Prec (%)	Rec (%)	F1 (%)
DenseNet-121	Speech	98.6	96.36	97.47
	Song	92.44	94.55	93.48
	Music	93.18	93.18	93.18
	Macro Avg.	94.74	94.7	94.71
DenseNet-v1	Speech	98.20	99.09	98.64
	Song	94.98	94.55	94.76
	Music	94.52	94.09	94.31
	Macro Avg.	95.90	95.91	95.90

Prec Precision, Rec recall, F1 F1-score.

**Table 5:** The time required for DenseNet-121 and DenseNet-v1 to predict a sample.

Model	Time Spent (ms)	Model Size (MB)
DenseNet-121	191.3	37.6
DenseNet-v1	123.5	2.9

Test platform was Qualcomm Snapdragon 662.

## Data Availability

Not applicable.
